# What influences on their professional development do general practice trainees report from their hospital placements? A qualitative study

**DOI:** 10.1080/13814788.2023.2191947

**Published:** 2023-05-03

**Authors:** Joanna Peart, Nele R. Michels, Karena Hanley, Cian Dolan, Julie Luyckx, Valerie Tanghe, Emma Peeters, Milda Burneikaite, Sonata Varvuolyte, Vesna Homar, Lucija Galič, Kamala Klobučar Kragelj, Geoff McCoombe, Nynke Scherpbier

**Affiliations:** a Irish College of General Practice, Dublin, Ireland; b Department of Family Medicine and Population Health, University of Antwerp, Antwerp, Belgium; c Faculty of Medicine, Clinic of Internal Diseases, Family medicine and Oncology, Institute of Clinical Medicine, Vilnius University, Vilnius, Lithuania; d Department of Family Medicine, Faculty of Medicine, University of Ljubljana, Ljubljana, Slovenia; e Department of General Practice and Elderly Care, Groningen University, Groningen, The Netherlands; f School of Medicine, University College Dublin, Dublin, Ireland

**Keywords:** GP/Family medicine training, professional development, professional identity, supervision

## Abstract

**Background:**

The clinical learning environment is important in GP specialty training and impacts professional development. Uniquely for GP trainees, about half of their training periods occur in a hospital environment, which is not their final workplace. There is still little understanding of how hospital-based training influences GP's professional development.

**Objectives:**

To seek the views of GP trainees on how their hospital experience contributes to their professional development as a GP.

**Methods:**

This international and qualitative study seeks the views of GP trainees from Belgium, Ireland, Lithuania, and Slovenia. Semi-structured interviews were performed in the original languages. A joint thematic analysis in the English language resulted in key categories and themes.

**Results:**

From the four themes identified, GP trainees were found to experience additional challenges on top of the service provision/education tensions, which are common to all hospital trainees. Despite these, the hospital rotation component of GP training is valued by trainees. A strong finding of our study is the need to ensure that learning from the hospital placements is placed firmly in the context of general practice, e.g. GP placements prior or parallel with the hospital placements, educational activities resourced by GPs during their hospital experience, encouraging hospital teachers to have greater awareness of the educational needs of GPs, including an awareness of their training curriculum.

**Conclusion:**

This novel study highlights how hospital placements for GP trainees could be enhanced. Further study could be broadened to recently qualified GPs, which may uncover new areas of interest.

KEY MESSAGES

- The trainee sees the hospital component of GP training as valuable.
- Quality of clinical supervision significantly influences the worth of hospital experience.
- Structuring the context of general practice during hospital placements, e.g., weekly release to GP training hours, maximises their value to the trainee.

## Introduction

The clinical learning environment has been described as the foundation of postgraduate medical education [1], with the quality of the training environment correlating to the later quality of care provided by graduates [2]. The challenges of providing such training in hospitals, in addition to the primacy of patient need and service provision, are well described [3,4]. Uniquely for General Practice (GP) trainees, some of this training is in an environment that is not their final workplace, i.e. the hospital, which furthermore may have little understanding of the future professional life of the GP trainee [5,6]. However, the hospital environment has been reported as providing a high prevalence of morbidity to assist GP trainees in learning, and to show future GPs what the hospital can provide in future care collaboration [7]. This model of GP training is standard across Europe (Table 1).

**Table 1.** Duration of GP training in EU countries.

Country	Hospital	Community	Total
	Years	Years	Years
Belgium	0–0.5	2.5–3	3
Denmark	2.5	2.5	5
Finland	0–2	4–6	6
Greece	3	1	4
Iceland	3	2	5
Ireland	2	2	4
Lithuania	1.5	1.5	3
Norway	1	4	5
Portugal	2	2	4
Slovakia	3–4	1–2	5
Slovenia	2	2	4
Spain	2	2	4
Sweden	1.5–2.5	2.5–3.5	5
Switzerland	2	2	4

The rise of competency-based medical education [8] emphasises the workplace as a learning environment [9–11]. Earlier publications on the hospital training component for GP trainees have focused on what GP trainees could learn from individual hospital placements [12–14]. While the hospital provides experience in the management of acute illness, technical practice, diagnostic procedures [5], in contrast, tolerating uncertainty, awareness of psychosocial factors and patient-centeredness are learnt well in the GP learning environment [15]. In addition, professional identity formation is now viewed as an essential aspect of specialty training [6,13,14], which may be more challenging for GP trainees in the hospital environment where there is occasional denigration of GP or undermining of GP trainees by some hospital specialists [5,15,16]. On the other hand, GP trainees have also reported good peer support in the hospital training and rated hospital paediatrics and emergency medicine as useful [17,18].

This international qualitative project seeks the views of GP trainees of how their hospital experience contributes to their professional development as a GP.

## Methods

A multi-country qualitative study, utilising semi-structured interviews, was undertaken. The research group consisted of GP trainees and supervisors from Belgium, Ireland, Lithuania, and Slovenia. Ethical approval was granted (or waived) by the appropriate body in the four countries. Belgium; Antwerp University Hospital (20/46 606), Ireland; The Irish College of General Practitioners (ICGP_REC_2020_T15), Lithuania; Not required, Slovenia; Republic of Slovenia Medical Ethics Committee (0120-381/2020/11).

### Developing the topic guide

Following a literature search, nominal group technique was conducted with international educators in a workshop delivered at the WONCA Europe Conference (Berlin 2020) [19]. Results were used to develop the topic guide (Supplementary Appendix 1) in English, then translated into Slovenian, Dutch and Lithuanian languages.

### Recruitment

Study participants (Table 2) were selected by purposeful samplng

**Table 2.** Participant demographics.

		Belgium	Ireland	Lithuania	Slovenia	Total
Gender	Female	12	4	11	4	20
	Male	6	5	3	2	13
Year of training	1st		4		1	5
	2nd	14	1	9	3	18
	3rd	4		5		4
	4th		4		2	6

to seek a broad range of trainees of different age, gender, prior experience, and country of primary medical degree.

Participation was invited through young doctor’s associations, national GP trainee databases, GP trainee social media groups, National Trainee Conferences, and Day Release teaching sites. GP trainees who had less than three months hospital experience were excluded.

### Data collection

Nine researchers (GP trainees from Belgium, Ireland, Lithuania, and Slovenia, 1 male and 8 female) conducted the interviews, which were face-to-face or *via* Zoom^®^. The interviews took place between January 2021 and May 2021 and were conducted in the language of GP training in that country. The interviews were recorded and transcribed (by hand or using Otter^®^ software). The transcripts were anonymised, stored according to European General Data Protection Regulation (GDPR) and imported into NVivo® software for analysis. Interviews continued until no further new information was forthcoming.

### Data analysis

Thematic analysis, following a six-step process [20], was employed to identify themes and patterned meanings. Data familiarisation of the transcripts and line-by-line open coding of each transcript was conducted by two researchers in each country, supported by NVivo^®^ (version 12). Initial meetings in each country discussed and refined the codes. Each country’s codebook was translated into English. Meetings with the researchers from all the countries condensed the codes and identified key categories and themes. Findings were verified using reflective conversations, comparing and contrasting the codebooks, noting and revising the categories in the light of the research question over several meetings, in line with previously published analytic methods [21,22].

### Reflexivity statement

Most researchers are career GPs (range of experience between 1-31 years). Some researchers had previously embarked on a career as a hospital specialist but had changed careers. One of the researchers is on a hospital medicine career. We remained aware that as a research group we may have had a vested interest in promotion of GP as a career and diminution of hospital medicine, and despite our best efforts to the contrary, our interpretation may be biased.

## Results

A total of 43 GP trainees participated, Belgium 18, Ireland 9, Lithuania 14, and Slovenia 6. Participants spread over different years of training and gender was split Female: Male 1.5:1 (Table 2, Participant Demographics). Average interview duration overall was 29 min: Belgium 30 min, Ireland 37, Lithuania 45, and Slovenia 13. In coding, an overall average inter-rater reliability of 92.5% was achieved: Belgium 90%, Ireland 94%, Lithuania 94%, and Slovenia 86%.

Our analysis revealed four themes: 1) supervision, 2) teaching, 3) tension between service delivery and learning, and 4) differing secondary care/primary care paradigms. Illustrative quotes, referred to in the theme discussion, are in Quotations Tables 3–6.

**Table 3.** Quotes, supervision.

	Sub Theme	Country	Quote	Gender	Year of training
	Approachability				
3a		Slovenia	The most valuable experiences were the ones where I was treating patients together with my supervisor which gave me confidence in treating similar cases on my own.	Male	2nd
3b		Belgium	If I raised the alarm, which I almost never did and said that I didn’t feel comfortable about a patient, they always came along. When I asked for help, I always got it	Female	2nd
3c		Ireland	Even on call like… just absolutely no pressure calling [the Consultant] or not… So it’s it’s, you feel really well supported, you know, and then you have more confidence in yourself	Male	2nd
3d		Ireland	You feel really well supported, you know, and then you have more confidence in yourself. Because you know that they’re there to back you up where as if you feel like you’re on your own, well, you start doubting yourself, you know, its mad, even though the decision is the same	Male	2nd
3e		Belgium	The Hospital Team told us ‘We know that you are a GP trainee and not an emergency physician in training. So as soon as you feel that something is too much, then you come to us’. They were true to their word by always being there when something was wrong	Female	3rd
3f		Ireland	The consultants were always very, very approachable, that if you’d had issues concern, be it as a standalone SHO or BST, or myself as a GP, you could always flag it with them, chatted to them. They’re very nice, very helpful. So I don’t think there was ever a moment if you felt out of your depth or that you couldn’t approach them or ask questions, always supported. You know, even in clinics, very, very busy endocrine clinics, you’re always running everything past the consultants, they often saw the patients if you’re concerned, you could flag it. You were never left to your own devices or unsupported.	Male	2nd
	Availability				
3 g		Ireland	You were left to some really sick patients. When I look back on that, it really kind of gives me shivers	Male	2nd
3h		Ireland	I remember examining like a two month old and not having a clue how worried I should be. I know this child looks really well, and there’s nothing wrong with them. But they’re two months old. Am I supposed to be worried about that? You know, so I just thought, why this model of complete isolation?	Female	4th
3i		Belgium	The hospital placement was really unlivable. Even on the weekend, I was extremely tired. I really didn’t get anything done. I also worked with such a severe pathology and I often had to initiate palliative sedation. In my opinion, supervision did not always act correctly and I therefore did not feel good if I had to pass those orders on to the nurses. Emotionally and physically I was really exhausted.	Male	2nd
3j		Lithuania	In some rotations you are completely alone, especially during night shifts. Then you are trying to find help, trying to ask for help. But no one has time for you and your problems	Female	3rd
3k		Belgium	I have been so well received by the nurses, because they really made me feel like I was part of their team and that I could make myself useful to them. If something had to be done, they called me much more frequently than the consultant. So yes, that was nice.	Female	2nd
3l		Belgium	I was mainly supported by the other trainees/registrars?. They know how it works. That was a very big help. Eating together, we did the rounds together and stuff, that was a very big help.	Female	3rd
3m		Ireland	In ED, you probably do get a sense the consultants don’t really care about as much that kind of thing, they are more ‘Whatever’, I think leave you out to the Regs. But the Regs are also lovely and friendly. I don’t think they care or notice what you are. They’re happy just to help	Male	4th
3n		Slovenia	Most of the times I didn’t even meet my supervisor and they didn’t even know they were my supervisors.	Female	4th
3o		Slovenia	The transfer of knowledge occurred mostly from other specialty residents to me - the ones that were interested in it and that didn’t have too much workload.	Female	4th
	Peers				
3p		Ireland	There was one Reg, who was there for I think, a two-month locum. And he was the person I learned the most from. Because it’s just one person you need. You need one person in a post in a job that brings you and guides you. You don’t need more out of the GP training, you don’t need like a whole list of people	Female	4th
3q		Ireland	Whether that comes in the form of a good SpR or or a good registrar, just a good leader in general, maybe in that speciality more, certainly does not have to be a consultant	Female	4th
3r		Belgium	If I ask the paramedics questions, I get the explanation. You have to ask proactively, it’s not that they come and ask if I would like to follow along. If I say I'm interested in it, they’re 100% open to it.'	Male	2nd

**Table 4.** Quotes, teaching.

	Sub theme	Country	Quote	Gender	Year of training
4a		Ireland	There’s different journal clubs, guidelines, kind of things. And they all happen as rotas made on a weekly basis and, you know, everyone has rotated through teaching roles and attendance rolls under the future as well, that oversees everything. So it’s quite, it feels more like a training course than going to just go in to provide a service.	Male	1st
4b		Belgium	In the hospital I have little time to look up or read something, in the general practice there was more time. It could sometimes be a quiet day there and then you could read a guideline, the hospital is always busy.'	Female	2nd
4c		Ireland	there would be a case presentation. And there would be two different types, there might be journal and there might be a clinical case presentation…. And that I found was great because it was kind of bleep free so everyone after a while in hospital they knew not to bleep you or to bleep say that specialty from say nine to 10 because they knew we were having our meetings, you know bar if you were on call. And there was another even in Gynae when we did have the structured mornings. They helped at the start, I was so enthused, when em, every Monday and Wednesday, I think it was, we would have, say, a physical exam tutorial.	Female	4th
4d		Belgium	They [the Consultants] also actively asked about my learning goals and what I expected, so that was positive.	Female	2nd
4e		Ireland	When it was more day to day, GP focus teaching, it was fantastic	Female	4th
4f		Lithuania	It was very beneficial when supervisors case specifically explained what the General Practitioner could have done better and what would he/she will have to do later.	Female	2nd
4g		Belgium	They [the Consultants] also made a lot of time to discuss things. If I said 'what do you expect from me as a general practitioner and what should I do if I want to refer a patient to you’. Certainly the supervisors who had me under their care, they really took the time to sit together for an hour and then look at pathology by pathology 'if you see this in general practice, what should you do, what should you exclude? and when do you have to refer it’. That was really useful just to see what they expect from me that I can do so that I can deliver a patient on a platter. Then you know what those options are.	Female	3rd
4h		Ireland	[The formal curriculum] is very good for overview and keeping [in] your mind that what you’re supposed to be learning and providing that kind of overall structure that’s going to be there for four years	Male	1st
4i		Ireland	From an academic point of view, it makes sense to write down what are all the things that people or trainees should be exposed to…. I'm not sure that that exercise has translated into our real experiences on the ground. It’s almost like an abstract parallel document or a set of structures that have been devised to give people a sense of comfort that we’re covering what we should be covering. But the reality is, we’re dropped into these different rotations and you learn what you learn	Male	4th
4j		Belgium	I also notice that the seminars have become more enriching since there are GP trainees in the hospital because many people have been able to develop a more critical view. I do find it interesting to see how the perspective of the GP trainees, especially in differential diagnostics, has become much broader and that they have much more 'know how’ within a certain topic.	Female	2nd

**Table 5.** Quotes, tension between service delivery and learning.

	Sub theme	Country	Quote	Gender	Year of training
	Administration				
5a		Slovenia	I wish hospitals would adjust our rotations to our needs and not to their needs.	Female	4th
5b		Belgium	You have a lot of paperwork, a lot of administration. If I see patients 1/4 of my time, that’s a lot. The rest is all computer work. I absolutely do not like that, I would rather be in the general practitioner’s practice where I do clinical work and I actually see patients	Male	2nd
5c		Slovenia	A lot of times I didn’t have any clinical assignment but just administrative assignments which have to be done but don’t contribute to my knowledge.	Male	2nd
5d		Ireland	Some patches, I felt like I was kind of treading water, doing the time, doing the service kind of job	Female	4th
5e		Slovenia	In the hospital I was more or less just a workforce for writing discharge papers.	Female	4th
5f		Slovenia	In orthopaedics I was just doing admissions and if I hade some time left I could go to emergency room, but even there I wasn’t really welcomed.	Female	4th
5g		Belgium	In the morning I did ward rounds and in the afternoon I did outpatient clinic, while I actually also had to do the wards as well. I also had to new admissions from ER in the morning and afternoon. So I had absolutely no time to finish anything.	Male	2nd
	Staffing				
5h		Lithuania	During Surgery rotation I had to spend all my time assisting in surgeries because of the lack of Staff. It is interesting to see these surgeries, however it is not beneficial for my future work as a GP	Female	3rd
5i		Ireland	[Excessive weeks of night shifts were] detrimental to your training because you know, if you had to do a week of nights, you miss a week of days and rounding with the consultant and those sorts of things	Female	4th
5j		Belgium	COVID has made it difficult. On one hand, there is a very high workload. But on the other hand, I also have the feeling that because of this I was able to see less pathologies and maybe get less out of this internship. COVID patients are all the same. So clinically you don’t learn much. We kind of have two extremes, either it’s COVID COVID COVID or nobody dares to come and we don’t see much pathology.	Female	2nd
5k		Belgium	I'm working on the paediatric psychiatry department specifically on the eating disorders unit. I don’t think that such a specific discipline is an addition to my GP training. I actually have a lot of stress going back to the GP practice because I feel like I've forgotten everything.	Female	2nd
5l		Lithuania	During Ophthalmology rotation most of us only watched how injections into Eyeball are being performed	Female	2nd
	Rotation duration				
5 m		Ireland	Six months in Psych is well enough for a GP trainee I would say. So, so maybe, maybe that was a slightly missed opportunity, you know. It could have been half that time and then got in a stint of ObsGyn, perhaps that might have been more useful	Female	4th
5n		Lithuania	I think the duration of 2 weeks is bad choice for a rotation because no one is eager to teach you because you will be leaving soon	Female	2nd
5o		Slovenia	Actually you can get a lot out of a 1 month rotation, especially if you are placed in an outpatient office and if you are committed to learning.	Male	2nd
5p		Belgium	I think it is valuable that the hospital placement starts if you have already worked as a GP trainee in general practice and that the hospital placement does not take place at the start of the training. Because if you had that in the beginning, then you are actually back like an intern and you just think ‘ah yes this is normal, this is the normal world’ and that is not always the case.	Female	2nd

**Table 6.** Differing secondary care/primary care paradigms.

	Sub theme	Country	Quote	Gender	Year of training
6a		Belgium	In the beginning I was very critical of all the investigations in ER and I wondered if all this was necessary. For example, everyone with memory problems gets a brain CT, even if they had one a few months ago. I find that over the months I've gotten a bit more compliant with this as it becomes a bit of a habit, although I don’t think it’s always correct. I try to remain critical. I also think that this will be an adjustment again when I go back to the GP practice to have that diagnostic uncertainty again. In the hospital everything must be proven on paper, clinical diagnoses hardly exist there everything must always be investigated with a technical examination..	Male	2nd
6b		Lithuania	In the hospital you see your patients every day. You can see the disease dynamics, changes after treatment and different procedures.	Female	2nd
6c		Slovenia	General medicine is not some subspecialty, we just need to see and learn a bit of everything.	Male	2nd
6d		Slovenia	It’s not important to go into details of each specialty field but just to learn the basics.	Female	4th
6e		Slovenia	During rotations, we get the basic knowledge of specialist fields which is enough for general medicine.	Male	2nd
6f		Lithuania	But instead of help I got comments from the staff such as: why are you here? What are you doing here? Who you even are? You get shouted at because you stood at a wrong place	Female	2nd
6g		Belgium	I don’t think I could have changed anything. I do think that we can ask 'why do you do it like that?' Then you sometimes quote how we would do it in general practice and then they respond 'ah yes okay’, but they stay with their policies. They are open to it, but I don’t think that will change their mindset.'	Male	3rd
6h		Belgium	That was a really nice team. They were used to working with interns and GP trainees, so the whole team was adapted to ‘Someone is coming here. He can’t do everything, but we can ask a lot of them and we have to make sure that they are really included in this team and that they can keep themselves busy’. So that was super positive and it just facilitates learning. That you feel 'part of the team’.	Female	3rd
6i		Slovenia	In the hospital, I felt the importance of team work. Here you are always with other people, other residents, doctors, nurses, nurse helpers and other staff. In the hospital, I could learn teamwork and team management, especially during emergency situations. This is very important in any job	Female	2nd
6j		Belgium	My hospital placement was very close to where I will establish myself as a general practitioner, my network is now very extensive/broad. I know which doctors I can call for advice and which pathology I have to refer to which doctor.	Female	2nd
6k		Belgium	I'm really happy that I can be a GP again, because I miss things in the hospital that you have as a GP. I also notice that my personality and my way of doing things match much better with the GP life than with the hospital life. With colleagues, but also with patients and in terms of pathology. I have found the right job.	Female	2nd
6l		Ireland	So I wondered, I don’t know but I wonder whether I might feel differently about things if I had some GP experience before doing certain hospital rotations, whether you might be able to bring some different kind of perspective on things as you know, having done some GP, there’s a few things that I remembered from when I was in the ED and particularly thinking ‘Oh, that’s why that’s like that, that’s why you get letters like this, for instance, on these sorts of presentations’. So I wonder whether that might affect your training? But I mean, you know, it’s just something I thought of.	Male	2nd
6m		Belgium	I think it is valuable that the hospital placement starts if you have already worked as a GP trainee in general practice and that the hospital placement does not take place at the start of the training. Because if you had that in the beginning, then you are actually back like an intern and you just think ‘ah yes this is normal, this is the normal world’ and that is not always the case.	Female	2nd

### Supervision

The supervision experience was found to vary between rotations, a consistent finding across all countries [Table 3(3a–3n)]. Some supervisors seem uninterested [Table 3(3m)]. A sense of responsibility to provide teaching was lacking [Table 3(3n)]. Some hospital consultants appeared to be reluctant to consider what the future professional life of the GP trainee might be like [Table 3(3m)]. Other hospital doctors, such as registrars, could fill this supervisory role in an effective manner [Table 3(3o–3r)].

Approachability was seen as one of the most important attributes of the supervisor [Table 3(3a)]. This was present when the trainee felt that questions on clinical matters were welcomed [Table 3(3b–d)], when the trainee felt safe that he/she will not be ridiculed and felt valued as a GP trainee [Table 3(3e)].

Availability of supervision was highlighted as a significant issue in the hospital environment with concerns for patient safety and trainee well-being as well as training quality. In Ireland and Lithuania, night shifts were noted to have dramatically reduced staffing with a resulting impact on clinical confidence compared to the daytime [Table 3(3j)].

GP trainees described many positive experiences. They reported being allowed to push themselves to make clinical decisions with back up [Table 3(3a–3d)] and opportunities to acquire experience in best clinical practice [Table 3(3a)]. They described a rich learning environment from peers [Table 3(3o–3r)] and being stimulated to read up on clinical presentations [Tables 3(3n) and 4(4a)], useful to them as future GPs.

### Teaching

One-on-one teaching was particularly valued as teaching tailored towards the future career as a GP, e.g. clear guidelines on when and how the trainee as a future GP should refer a patient to the hospital [Table 4(4d–g)].

While each national GP training body or institution had a formal curriculum, hospital supervisors rarely referenced it, an experience noted across all countries [Table 4(4h)]. Sometimes the hospital rotations did not provide any opportunities for learning which matched the curriculum [Tables 4(4i) and 5(5a)].

Insufficient teaching was lamented in all countries [Table 5(5a)]. Regular assessment was considered lacking in Slovenia and Lithuania. In Ireland and Belgium, the GP trainees leave the hospital clinical environment once weekly for training from GP educators. This was felt to make the hospital experience more relevant [Table 4(4j)]. In Lithuania, seminars are delivered by GP educators on site in the hospitals during the hospital rotations. Slovenia has no dedicated GP training during the hospital rotations.

### Tension between service delivery and learning

Administrative work, such as discharge letters, was considered less valid to their training by GP trainees [Table 5(5b–5d)]. A unique finding in our study is that GP trainees felt they often shouldered a disproportionate amount of such service work to release the hospital specialist trainees on their team for clinical work [Table 5(5e,5f)].

GP trainees felt the sheer volume of work to be a hindrance to learning in all countries [Table 5(5g–5i)]. Conversely, on occasion, there was an insufficient number of patients and too many hospital doctors seeking experience [Table 5(5j)]. The range of expertise in some speciality wards could be narrow, limiting learning opportunities e.g. eating disorders or cataract surgical ward [Table 5(5k, 5l)]. Excessive on-call duties also hinder learning both in terms of time, by missing educationally richer day shifts, and fatigue levels [Table 5(5i)].

Interviewees demonstrated excellent insight into their training needs and the likely demands of their future role, stating that shorter specialised rotations would create an opportunity for other more relevant experience [Table 5(5m)]. General rotations, those with larger volumes of outpatient experience were mostly highly valued [Table 5(5h,5k,5o)] with some rotations thought to be of little value to a GP trainee at all [Table 5(5k,5l)]. The ability to tailor rotations to learning needs, such as in Slovenia, was limited in other countries by clashes with the logistics of service provision [Table 5(5e,5h)].

### Differing secondary care/primary care paradigms

GP trainees noted that approaches to patient care differed in the hospital environment compared to GP. Hospital-based care focuses on completing multiple investigations quickly in contrast to GP where these investigations can proceed more slowly, using time as a diagnostic tool [Table 6(6a)].

GP training is challenging due to the breadth of what needs to be learnt. This contrasts with the depth of knowledge required for specialist care in hospitals. Some GP trainees felt that their hospital experience immersed them in detail which was more than they needed to know for a future career in GP [Table 6(6c–6e)].

GP trainees valued learning how the hospital system works, providing insight into the patient’s journey on presentation from the emergency department through to the outpatient clinics, in addition to the clinical opportunity of seeing the course of an illness [Table 6(6b)]. Trainees also valued learning how to work as a team and building a future professional network [Table 6(6h–6j)].

GP placement early in GP training was beneficial, allowing the GP trainee to better self-direct their training to their future role [Table 5(5p)]. Some spoke of awareness of being ambassadors of General Practice while in their hospital clinical placement [Table 6(6k)]. Belgian GP trainees, placed in GP rotations before commencing the hospital part of their training, demonstrated a strong sense of GP identity during hospital rotations, presenting their view of what a GP approach to clinical care would be to hospital colleagues [Tables 4(4j) and 6(6g)]. An Irish interviewee specifically participated in the study to express how he felt having a GP placement prior to his hospital rotations would have enriched his hospital experience on several levels [Table 6(6l)]: by understanding the limitations of services available to a GP, by understanding better what a patient might need from a GP action, e.g. a referral to A&E, by giving greater insight into what was relevant for him to learn during his hospital experience and finally by providing an opportunity to educate his peers on the context of GP referrals to his hospital peers. This view was supported by a Belgian interviewee [Table 6(6m)].

Unfortunately, there were negative comments about how some hospitals perceived the contribution of GP trainees. In Lithuania. GP trainees were singled out as not belonging in the hospital, e.g. by derogatory comments from consultants or other hospital team members [Table 6(6f)]. This affected the trainee’s sense of being a team member [Table 6(6g)] and represents a missed opportunity for the positive relationships created between career hospital doctors and career GPs as noted in Belgium.

## Discussion

### Main findings

This qualitative study gives insights into the views of GP trainees from different European countries of how their hospital experience contributes to their professional development as a GP.

### Valued

It was clear that GP trainees valued their hospital-based training rotations despite the conditions experienced. This was most noted in Ireland and Belgium. This counters a previous argument by Goldie (23) for situating UK GP training entirely in General Practice.

### Identity dissonance

Cruess et al. (2018) recommend adopting the ‘communities of practice-theory’ as the overarching educational theory in medical education [24]. Each hospital department where a trainee is placed is a ‘community of practice’ and our research shows that these were not always the ideal training environment for GP trainees. Support of doctors in training should consist of an inclusive welcome to the community, access to activities appropriate to the level of the learner, instruction, role modelling and mentoring, and charting progress through assessment and feedback [25]. This is not consistently present during hospital rotations for GP Trainees.

### Unique learning opportunities

Some of the learning on hospital rotations, e.g. current best practice in a specialty, or the full range of presentations which can occur, could not have been learnt in GP. The hospital rotations supported the development of clinical confidence, learning how to work in teams and learning what happens to a patient admitted to hospital. Also, the hospital experience assists in formatting professional connections for those who would practice in the future as a GP in the locality.

### Context

An important finding in this study is the need to situate the learning from the hospital experience in the context of general practice. Contact with GP educational supervisors, either through off-site protected half-day release, or through GP rotations early in training, assisted identity formation as a GP and helped trainees use learning opportunities better. Belgian trainees described educating their hospital colleagues on primary care approaches. Cross-education between primary and secondary care by hospital-based GP trainees (with experience in General Practice) could be a valuable opportunity to deepen understanding of each other by both environments.

### Supervision

Quality of supervision is the most pivotal aspect affecting the value of the hospital rotation. In keeping with AMEE guidelines [26], the authors recommend that hospital supervisors be aware of the requirements of the training body, and supervision should be structured with regular timetabled meetings [27]. Based on the GP trainee’s comments, there is room for improvement in the quality of supervision for GP trainees on hospital placements across all countries.

### Strengths and limitations

Strengths of the study include the spread of data collection across four European countries with a range of investment of GP placement time within GP training, from countries with a high proportion (Belgium, Ireland) to countries with lower proportions (Slovenia, Lithuania).

Limitations of this study include that the interviews were conducted in four different languages and some distortion of the meaning may have occurred in translation. Individual country GP programmes can be limited by availability of training positions which gives significant heterogeneity and resulting experiences. Another limitation is that the subjects interviewed were all trainees. Widening participants to recently qualified GPs now working in General Practice may have uncovered more recognition of the differences between primary and secondary care. A further limitation is that the COVID-19 pandemic may have affected the responses in our data, as some of the more usual formal teaching was lost and so may be under-reported.

## Conclusion

This study shows that GP trainees valued their hospital experience, especially where approachability and availability of hospital teachers improves the quality of supervision. It uniquely shows that GP trainees have additional challenges as trainees in the hospital environment. These include an identity dissonance of being a GP trainee in the hospital environment, shouldering a greater service work administrative burden compared to their hospital specialty peers, and on occasion, being excluded from the community, which should support the learner.

## Supplementary Material

Supplemental Material
