# Supplementary material for: What influences on their professional development do general practice trainees report from their hospital placements? A qualitative study
Source: Eur J Gen Pract. 2023 May 3;29(1):2191947. doi: 10.1080/13814788.2023.2191947 (PMC10158549; doi:10.1080/13814788.2023.2191947)
Supplement: Supplemental Material [file IGEN_A_2191947_SM6053.docx]

Date:

Time:

Place:

Interviewer:

Interviewee:

INTRODUCTION

This study aims to discover the promoting and/or hindering experiences GP/FM trainees report on their professional development from their hospital placements.

The study objectives are:

- To explore the positive and/or negative experiences of GP trainees on their professional development in their hospital placements
- To ascertain which of these experiences have a greater impact on the trainee development
- To provide avenues for further research into improving the quality of training received by GP trainees from their hospital rotations
- To inform areas of development for training bodies to target to improve the provision of training

My name is [ ]

Confidentiality

- Audio recordings will be destroyed within a month of transcription
- A researcher independent of the interviewers will remove all identifiers off the scripts and give each script a unique identifier known only to them. This unique identifier will be maintained in an Excel database.
- Other researchers will subsequently analyse deindentified transcripts.
- Data will be retained for two years on a password-protected laptop.
- You may request a copy of your transcript up until the time that the data is destroyed
- You do not have to answer any question that you do not wish to
- You may stop the interview at any time without consequence to you
- Before commencing the interview, please verbally confirm your consent to participate
- Before commencing the interview, please verbally confirm your consent to this interview being audio recorded

QUESTIONS

Broad questions

- How is your current placement?
- Tell me broadly about your experience of hospital training as a GP trainee so far

In-depth questions

- Training experience:
  - What aspects of your hospital-based training (formal or informal) did you find most beneficial to your training as a future GP?
  - Were there any aspects of your hospital-based training, which adversely affected your training as a future GP?
  - Could you comment on the types of department/hospital speciality and their contribution to your professional development?
  - Could you comment on the length of the hospital placements and their contribution to your professional development?
  - Do you have any comments on the hospital placement(s) about handling uncertainty in General Practice?
  - (optional) Do you have any comments on the hospital placement(s) in relation to learning about collaboration?
- Trainee support
  - What were the best ways in which you were supported as GP Trainee in a hospital placement?
  - Are there any ways in which you felt unsupported as a GP Trainee in a hospital placement?
  - How did you experience the link made between your work in the hospital placement and your work as a future GP?
  - How do you see the balance between service provision and education in the hospital placements?
- Overall
  - At this stage of your training what has been the most influential aspect from your hospital training that has contributed, positively or negatively to your training so far?

Clarifications and link to theory

- You mentioned [ ], can you tell me more about that?
- You mentioned [ ], how did that impact your awareness?
- You touched on “XXX”, which other contributors have also mentioned. Is there anything more you would like to say on this?
- Is there anything relevant to this research area that you would like to bring to our attention?

CLOSING

This study will contribute to better understanding of how to organise GP training to benefit of future trainees in Ireland and across Europe.

Thank you for your interest and your time.

The recording of this interview will be transcribed using software Otter and analysed using NVivo software. This is expected to take 11 months. After that it will be synthesised with interviews from our partner countries.
